# Cost utility of a pharmacist-led minor ailment service compared with usual pharmacist care

**DOI:** 10.1186/s12962-020-00220-0

**Published:** 2020-07-28

**Authors:** Sarah Dineen-Griffin, Constanza Vargas, Kylie A. Williams, Shalom I. Benrimoj, Victoria Garcia-Cardenas

**Affiliations:** 1 Graduate School of Health, University of Technology Sydney, Sydney, NSW Australia; 2 Centre for Health Economics Research and Evaluation, University of Technology Sydney, Sydney, NSW Australia; 3 University of Granada, Granada, Spain

**Keywords:** Cost utility, Cost effectiveness, Minor ailment services, Self-care, Community pharmacy, Community pharmacy services, Health services

## Abstract

**Background:**

A cluster randomised controlled trial (cRCT) performed from July 2018 to March 2019 demonstrated the clinical impact of a community pharmacist delivered minor ailment service (MAS) compared with usual pharmacist care (UC). MAS consisted of a technology-based face-to-face consultation delivered by trained community pharmacists. The consultation was guided by clinical pathways for assessment and management, and communication systems, collaboratively agreed with general practitioners. MAS pharmacists were trained and provided monthly practice support by a practice change facilitator. The objective of this study was to assess the cost utility of MAS, compared to UC.

**Methods:**

Participants recruited were adult patients with symptoms suggestive of a minor ailment condition, from community pharmacies located in Western Sydney. Patients received MAS (intervention) or UC (control) and were followed-up by telephone 14-days following consultation with the pharmacist. A cost utility analysis was conducted alongside the cRCT. Transition probabilities and costs were directly derived from cRCT study data. Utility values were not available from the cRCT, hence we relied on utility values reported in the published literature which were used to calculate quality adjusted life years (QALYs), using the area under the curve method. A decision tree model was used to capture the decision problem, considering a societal perspective and a 14-day time horizon. Deterministic and probabilistic sensitivity analyses assessed robustness and uncertainty of results, respectively.

**Results:**

Patients (n = 894) were recruited from 30 pharmacies and 82% (n = 732) responded to follow-up. On average, MAS was more costly but also more effective (in terms of symptom resolution and QALY gains) compared to UC. MAS patients (n = 524) gained an additional 0.003 QALYs at an incremental cost of $7.14 (Australian dollars), compared to UC (n = 370) which resulted in an ICER of $2277 (95% CI $681.49–3811.22) per QALY.

**Conclusion:**

Economic findings suggest that implementation of MAS within the Australian context is cost effective.

*Trial registration* Registered with Australian New Zealand Clinical Trials Registry (ANZCTR) and allocated the ACTRN: ACTRN12618000286246. Registered on 23 February 2018.

## Background

Minor ailment presentations to emergency departments (EDs) and general practitioners (GPs) for conditions such as headaches, coughs, colds and earaches are an inefficient use of public resources [1]. Minor ailments have been defined in the literature as “*self*-*limiting or uncomplicated conditions that can be diagnosed and managed without medical (i.e. GP) intervention*” [2–4]. Self-care is the preferred method of managing minor ailments for many patients [5]. A 2019 policy statement from the International Pharmaceutical Federation and the Global Self-Care Federation, described the intention of the pharmacy profession to facilitate self-care and further develop self-care as a “pillar of sustainable health systems” [6, 7]. This statement supports pharmacists to encourage consumers to use health system resources responsibly and engage in self-care when appropriate [6, 7].

Internationally, governments have been investing in supporting pharmacies to enhance self-care and self-medication practices [8–10]. Health policies have been developed in a number of countries to encourage self-care at the community pharmacy level. This is probably due to increases in GP and ED presentations, which has led governments to review policy to support self-care [10–15]. Minor ailment services (MASs) in the United Kingdom (UK) and pharmacists prescribing for minor ailment (PPMA) services in Canada have been implemented in community pharmacies [16]. Other countries, such as Spain [17], New Zealand [18] and Ireland [19] are evaluating the feasibility of similar initiatives.

Several arguments or reasons have been proposed for the development of health policies encouraging health systems to incorporate this type of services, some being focused on reducing the load/number of visits to general practice and emergency settings. Others have focused on the economic aspects of transferring the management of minor ailments to the community pharmacy setting. In this regard, there are a number of economic studies published in the literature focusing on community pharmacist management of minor ailments in the UK and Canada [3, 16, 20–27]. In the UK Watson et al. estimated the cost and health outcomes of pharmacy-based care of minor ailments compared with care provided in general practice and ED settings, using a prospective cohort study design [25]. Mean overall costs per consultation were £29.30 for pharmacy care, when compared with general practice (£82.34) and ED (£147.09) [25]. As pharmacy was estimated to be less costly and as effective (in terms of symptom resolution) compared with ED and general practice, it was said to dominate both of these options [25]. Similarly, Rafferty et al. conducted an economic impact analysis measuring the costs of pharmacists prescribing for minor ailments, and the alternative scenario of usual care in Canada [16]. These studies show a positive economic impact through reduced costs associated with the unnecessary use of other more expensive health services and settings for the management of minor ailment presentations [25]. Although the international literature is positive, application of MAS to the Australian health system requires local data to ensure transferability. No economic evaluations have been conducted to date using local data of a community pharmacist-delivered service (MAS) compared with usual pharmacist care (UC), which represents current practice in Australia. This would allow a better understanding on the costs and outcomes associated to this intervention and ultimately assess if MAS represents a value for money intervention in the Australian setting.

## Methods

### Economic analysis description

The economic evaluation consisted of a cost utility analysis (CUA) (Table 1). Direct health care costs affecting both, the health care system and the patient through out of pocket costs, were included in the analysis. Although broader cost implications to the society (e.g. indirect costs) were not taken into consideration, the perspective was considered as a societal as some of the direct health care costs were borne by actors outside of the health care system, the patient [28]. By definition, a minor ailment is a self-limiting problem and implicitly involves resolution regardless of the intervention performed by the pharmacist [29]. A time horizon of 14 days was considered appropriate to account for costs and health outcomes given minor ailments are generally time limiting and would resolve in this time frame. A 14-day time horizon has been previously applied in international studies assessing minor ailments and symptom resolution [25]. Furthermore, the time frame was pragmatically chosen by researchers to reduce the possibility of recall bias [30]. Costs were measured in 2018 Australian dollars (AUD) and health outcomes were measured in quality adjusted life years (QALYs). Deterministic and probabilistic sensitivity analyses were also conducted to account for robustness and uncertainty of the results. The analyses’ are reported according to the Consolidated Health Economic Evaluation Reporting Standards checklist [31].

**Table 1:** Components of the economic evaluation

Types of analysis	CUA
Intervention	Pharmacist-led minor ailment service (or MAS)
Comparator	Usual pharmacist care (or UC)
Outcomes	QALY Episode of appropriate pharmacist care Extra patient achieving symptom resolution
Perspective	Societal
Time horizon	14 days
Method used to generate results	Decision tree
Software	Microsoft Excel for Mac Version 16.16.10

*CUA* cost utility analysis, *QALY* quality adjusted life years

### Decision tree model

**Fig. 1:**
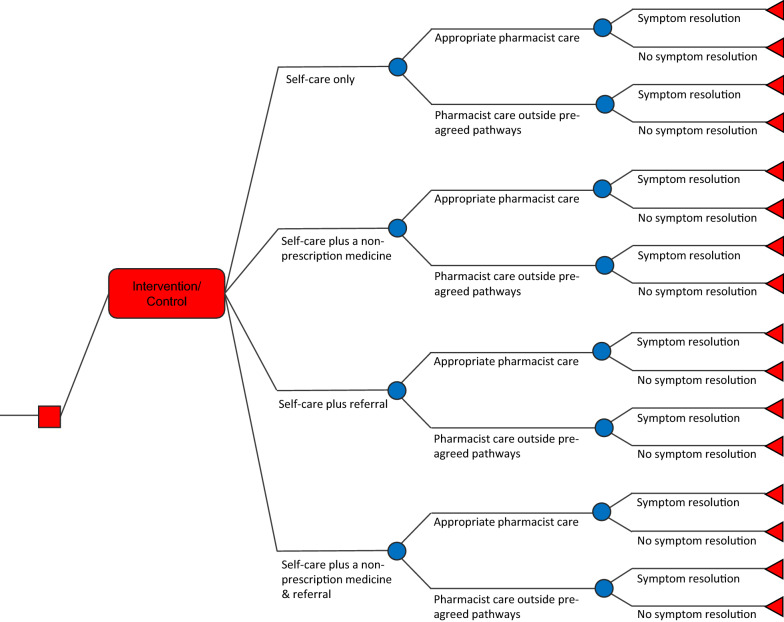
Decision tree model structure. *MAS* minor ailment service, *UC* usual pharmacist care

A decision analytic modelling technique was employed for the economic evaluation which consisted of a decision tree implemented in Microsoft Excel for Mac Version 16.16.10 (Fig. 1). A decision tree was considered the most adequate modelling technique because the decision problem surrounding minor ailments is relatively simple and straight forward (a once-only disease event); a limited number of health states were identified as relevant; the short model duration; and the fixed time horizon was pre-specified. As depicted in Fig. 1, the two strategies (MAS and UC) are denoted by each branch from the initial decision node (square in Fig. 1). ‘Appropriate pharmacist care’ is an intermediate outcome measure for cost-effectiveness (a proxy for health gain) and is defined as “the provision of appropriate self-care, non-prescription medicines and/or medical referral in line with the pre-agreed management pathway (HealthPathway) for each minor ailment” [32]. The terminal node represents the end point of the patient pathway whereby patients achieve symptom resolution or not within 14 days following the initial interaction with the pharmacist (triangle in Fig. 1) [33]. This structure allows comparison of the expected costs and outcomes of the two alternative pathways. The difference in probabilities, costs and quality of life were generated to derive the total incremental impact of MAS, compared with UC, in a cohort who received: (i) appropriate pharmacist care and achieved symptom resolution; (ii) appropriate pharmacist care and did not achieve symptom resolution; (iii) pharmacist care outside of the agreed pathways and achieved symptom resolution, or (iv) pharmacist care outside of the agreed pathways but did not achieve symptom resolution.

The model was populated with probabilities and costs obtained from a cluster randomised controlled trial (cRCT) undertaken in Australia between July 2018 and March 2019 (https://www.anzctr.org.au identifier: ACTRN12618000286246) evaluating the effectiveness of MAS, compared to UC [34, 35]. Sites recruited were community pharmacies in the region covered by the primary health network (PHN) of Western Sydney [36] and were randomised using a computer-generated random number list (ratio of 1:1) in Microsoft Excel for Mac Version 16.16.10. Eligible patients were recruited by a study pharmacist, from one of thirty pharmacies, if: (i) aged 18 years or over; (ii) requesting or self-selecting a medicine to treat symptoms (product-based presentation) and/or directly asking for pharmacists advice related to their symptoms (symptom-based presentation) for one of the following conditions: reflux, cough, common cold, headache (tension or migraine), primary dysmenorrhoea, and back pain; (iii) attending the pharmacy in person; (iv) able to provide consent; and (v) contactable by telephone. Patients received MAS or UC depending on allocation of the pharmacy to which they attended. Data in both arms was collected and managed using the Research Electronic Data Capture (REDCap) tool on provided iPads [37]. Pharmacists completed a data form for each patient. Patients were contacted at 14-days by phone to complete a follow-up questionnaire. Full details of the intervention and study protocol have been previously published [29, 34, 35].

#### Intervention (MAS)

There were four features as part of the intervention which included:

1. Standardised consultation

Patients received a face-to-face technology-based consultation with a trained community pharmacist specific to their ailment. Pharmacists followed a number of steps in the patient encounter, including:

- Service offering, during which the pharmacist explained the features of the service.
- Clinical assessment, where the pharmacist elicited relevant clinical information and checked for referral symptoms.
- Standardised management, where the pharmacist utilised agreed pathways, which included the provision of self-care, non-prescription medicine(s) where appropriate, and/or referral to another healthcare provider.
- Documentation, where the pharmacist documented patient data in a data collection form. The pharmacist sent a direct massage to the patients’ GP with details of the consultation (with patient consent) using HealthLink [38].

To deliver the standardised consultation, MAS group pharmacists were provided with:

1. Integrated technology platforms agreed with GPs
  HealthPathways [32]: Evidence-based pathways specific to each ailment. The research and writing of each pathway followed a literature review of international and national guidelines with leading GPs involved in the PHN governance. The pathways were used by pharmacists to guide their assessment and management during each consultation.
  HealthLink [38]: A secure messaging system allowing bidirectional communication between the community pharmacist and the GP.
2. Educational training program for pharmacists

Pharmacists delivering MAS were trained for 7.5-h by researchers and GPs at Western Sydney PHN. Training aimed to ensure pharmacists competency in delivering MAS, clinical areas, consultation skills, recognising red flags referral criteria, documentation processes and technology systems. The workshop involved a combination of lectures and interactive sessions.

(3) Practice change support for pharmacists

Pharmacists delivering MAS were provided 1-h monthly visits consisting of practice support and on-site training by a practice change facilitator (PCF). The PCF monitored data quality and intervention fidelity. PCFs were trained to ensure these objectives were met. PCFs addressed barriers to practice change using evidence-based strategies and collected both quantitative and qualitative data.

#### Comparator (UC)

Patients attending the UC group of pharmacies received usual pharmacist care on presentation to the pharmacy. Pharmacists in the UC group did not receive any of the educational interventions outlined above including the support of a practice change facilitator. They attended a 2-h training workshop on data collection systems and recruitment at Western Sydney PHN.

### Model input parameters

#### Transition probabilities

Transition probabilities to the defined health states (Fig. 1) were directly derived from our cRCT study data, regardless of whether ‘appropriate pharmacist care’ was provided during the patient-pharmacist encounter. It was assumed that patients reporting partial resolution of symptoms at follow-up would achieve complete resolution given the self-limiting nature of minor ailment conditions [39].

#### Costs

Costs were measured using cRCT trial data and valued using local sources [29]. There were a number of costs identified and estimated. A pharmacists’ average hourly rate was sourced from the Pharmacy Industry Award [40] and was multiplied by the time consumption to deliver MAS or UC. Costs of non-prescription medicines (paid for by the patient) were determined by averaging the price of medicines across three pharmacy groups including Chemist Warehouse, Amcal and Priceline. Reconsultation and referral costs consisted of costs of contacts with other health care providers. Costs were included for patients who had: (i) adhered to pharmacist’s referral (adherence was established at follow-up by confirming whether the patient had reported visiting their healthcare provider within the 14-days following the consultation) or; (ii) reconsulted with a medical provider (reconsultation was established at follow-up for patients not referred by the pharmacist, but had reported seeking care from another health provider within 14-days following the consultation). The average cost of a GP consultation was determined through examination of Medicare Benefits Schedule (MBS) reports. Referral adherence and reconsultation costs were calculated by considering the average cost per consult and patient out-of-pocket costs for all medicines (including non-prescription and prescription products). Prescription prices were determined using Pharmaceutical Benefits Scheme (PBS) and non-PBS prices for individual items and the total number of medicines per patient. A cost for training, technology and facilitation was included for the MAS arm only. Training costs were included for the pharmacists who received MAS training. This was calculated by multiplying the pharmacists’ average hourly rate by total training time. The costs of workshop facilitators and all training materials were incorporated. Costs for monthly visits to MAS pharmacies by the practice change facilitator were also included. A facilitation cost was determined by multiplying the hourly rate of the practice change facilitator, by the total number and duration of visits to MAS pharmacies. Technology costs including an annual license cost per pharmacist for secure messaging software, were included. An estimated number of patients per pharmacy, based on industry data, [41] was used to estimate the average cost ‘per patient’ for training, facilitation and technology.

#### Utilities

Utility values were not available from our cRCT study data, hence we relied on utility values reported in a 2015 study (MINA study) conducted across two geographic regions in the UK (East Anglia, England and Grampian, Scotland) [25]. The study was an observational study, with a prospective cohort design, carried out over a time horizon of 14-days. The aim of the study was to compare the effectiveness (patient outcomes) and cost-effectiveness of care for selected minor ailments across a number of settings including community pharmacy, general practice, and ED [25]. The study examined multiple clinical conditions including musculoskeletal aches and pains in arms, legs, back, hands and feet, eye discomfort, nausea, vomiting, diarrhoea, constipation, sore throat, cough, cold and sinus. Costs were estimated for the initial consultation and any actions taken in the following two-weeks for each minor ailment (including health care contacts, medication and investigations). To estimate the impact of the index consultation on health-related quality of life, participants were asked to complete the EuroQoL EQ-5D-3L at baseline and at 2-week follow-up [25] (Table 2).

**Table 2:** Utility values for pharmacy based minor ailment care

EQ-5D-3L utility follow-up	Mean utility (SD)	Reference
Symptom resolution	0.91 (0.15)	Refer to MINA study [25]
No symptom resolution	0.77 (0.22)	

The total accrued QALYs for each arm in the model were estimated by calculating the area under the curve (the time spent (14 days) in each health state (symptom resolution or no symptom resolution), multiplied by the corresponding utility value). QALYs were considered an adequate outcome measure as it allows comparability across different diseases and interventions, provides a common metric for comparing cost per unit of health gain and the outcome measure recommended in Australia.

Results of the CUA are presented as the incremental cost-effectiveness ratio (ICER), calculated by dividing the difference in total accrued costs (incremental cost) by the difference in the total accrued QALYs (incremental effect) [42]. The ICER was considered against a willingness-to-pay threshold. While Australia has not yet defined an explicit willingness-to-pay threshold, a base-case reference threshold of $28,033/QALY (95% CI $20,758–37,667) has been recommended [43].

### Sensitivity analyses

#### Deterministic SA

A one-way deterministic SA was conducted to assess the impact of all known individual parameters tested independently, *ceteris paribus*, applying upper and lower limits owing to changes in assumptions made for the base-case analysis. The results for this analysis were summarised in a tornado diagram which shows the varying effects on the overall ICER. In addition, a multi-way SA was conducted to determine the impact of simultaneous changes to multiple parameters on the ICER. This was conducted to assess the extent the results may vary assuming a worst-case scenario analysis. The worst-case scenario was defined as one with the highest possible patient-pharmacist consultation cost and assumes all patients adhere to referral recommendations made by the pharmacist.

#### Probabilistic SA

A probabilistic SA was conducted by applying Monte Carlo simulation [44]. The model was made probabilistic in order to account for joint parameter uncertainty. Individual parameters were assigned a parametric distribution, assuming a homogeneous sample of patients, to inform the parameter estimation (Table 3) [45]. The results were used to estimate the probability that MAS is cost-effective. The analysis was run in Microsoft Excel for Mac Version 16.16.10 software. The results are presented as a cost-effectiveness plane (scatter plot) and an acceptability curve.

**Table 3:** Parameters used to populate the economic model and distributions for uncertainty analysis

Health resource	Mean model value	Std. error	Minimum	Maximum^a^	Source	Parametric distribution (PSA)
Costs						
Pharmacist rate (per hour)	$29.37	$2.52	$24.04	$34.30	Australian Government Fair Work Ombudsman 2018 [40]	Gamma
Time to deliver MAS (minutes per patient)	10.88	0.18	10.52	11.23	cRCT data [29]	Normal
Time to deliver UC (minutes per patient)	3.29	0.21	2.88	3.71	cRCT data [29]	Normal
Trainings with MAS (number per year)	1	0.51	0	2	cRCT data [29]	Normal
Facilitator rate with MAS (per hour)	$46.28	$4.72	$37.02	$55.54	University of Technology Sydney award level HEW5 Step 1; cRCT data [29]	Gamma
Facilitator visits with MAS (per month)	1	0.51	0	2	cRCT data [29]	Normal
Average training, facilitation and technology cost with MAS (per patient)	$0.07	$0.02	$0.00	$0.11	Purchase invoices; cRCT data [29]	Gamma
Average nonprescription medicine price with MAS (per patient)	$10.62	$0.22	$10.20	$11.05	Amcal, Chemist Warehouse, Priceline 2019 data; cRCT data [29]	Gamma
Average nonprescription medicine price with UC (per patient)	$9.76	$0.20	$9.39	$10.14		Gamma
Average cost of medicines at reconsult (per patient)	$9.79	$0.94	$7.94	$11.64	PBS 2019; Amcal, Chemist Warehouse, Priceline 2019 data; cRCT data [29]	Gamma
General practitioner fee (per consult)	$44.07	$6.74	$30.85	$57.29	MBS 2019 [46]	Gamma
Probabilities						
Symptom resolution (MAS)	0.75	0.02	0.73	0.77	cRCT data [29]	Beta
Appropriate pharmacist care (MAS)	0.87	0.01	0.85	0.88	cRCT data [29]	Beta
Symptom resolution (UC)	0.74	0.02	0.65	0.69	cRCT data [29]	Beta
Appropriate pharmacist care (UC)	0.68	0.02	0.71	0.76	cRCT data [29]	Beta
Utilities						
Symptom resolution	0.91	0.02	0.88	0.94	Refer to Watson study [25]	Beta
No symptom resolution	0.77	0.02	0.73	0.81		Beta

*HEW* higher education worker, *MAS* minor ailment service, *MBS* Medicare Benefits Schedule, *PBS* Pharmaceutical Benefits Scheme, *PSA* probabilistic sensitivity analysis, *UC* usual pharmacist care

^a^Lower and upper bound values represent 95% confidence interval; or upper and lower range from trial data

## Results

Eight hundred and ninety-four patients (n = 894) were recruited by thirty pharmacies (n = 30) in the cRCT. Eighty-two percent (n = 732) of patients were followed up at 2-weeks. Both effectiveness (patient outcomes) data and costs were available.

### Model input parameters

Table 3 outlines the model input parameters and sources used to populate the economic model.

Table 4 shows a summary of the estimated mean costs for each cost category in both arms. The descriptive results show the primary difference in mean cost per patient arises from consultation time and referral adherence (due to the higher referral rate and adherence to referral seen with MAS).

**Table 4:** Estimated mean cost of MAS and UC

	Mean cost per patient ($AUD)^a^	
	MAS	UC
Consultation time	5.33	1.61
Non-prescription medicine	10.85	10.36
Referral adherence (including medicines)	5.59	0.61
Reconsultation (incl. medicines)	7.73	9.70
Training, facilitation, technology set-up	0.07	–
Total cost (SD)	29.56 (4.21)	22.28 (4.59)

*AUD* Australian dollars, *MAS* minor ailment service, *UC* usual pharmacist care

### Economic analyses

Total cost and outcomes, along with incremental cost and incremental outcomes are shown in Table 5. On average, MAS was more expensive but also more effective compared to UC. With respect to the QALYs, patients (n = 524) receiving MAS gained an additional 0.003 QALYs at an incremental cost of $7.14, compared to UC (n = 370). The results indicate an ICER of $2277 (95% CI $681.49–3811.22) per QALY. For the clinical effect measure of appropriate pharmacist care, the service resulted in an incremental score of 0.191 additional patients receiving appropriate pharmacist care, relative to UC, resulting in an ICER of $37.42 per additional episode of appropriate pharmacist care. For the clinical effect measure of symptom resolution, MAS resulted in an incremental score of 0.012 additional patients achieving symptom resolution, relative to UC, resulting in an ICER of $586.88 per additional patient achieving symptom resolution.

**Table 5:** Incremental analysis: Cost-utility (base case) and cost-effectiveness results

	Mean cost per patient (SD)	Total outcome	Inc. cost (AUD)	Inc. outcome	ICER (AUD/outcome)
Outcome = QALY					
UC	$19.75 (SD $7.47)	0.0264			
MAS	$26.88 (SD $7.62)	0.0296	$7.14	0.003	$2277
Outcome = episode of appropriate pharmacist care (care meeting agreed treatment pathways)					
UC	$19.75 (SD $7.47)	0.676			
MAS	$26.88 (SD $7.62)	0.866	$7.14	0.191	$37.42
Outcome = extra patient achieving symptom resolution					
UC	$19.75 (SD $7.47)	0.738			
MAS	$26.88 (SD $7.62)	0.750	$7.14	0.012	$586.88

*AUD* Australian dollars, ICER incremental cost effectiveness ratio, *MAS* minor ailment service, *QALY* quality adjusted life year, *UC* usual pharmacist care

The costs used in the cost utility and cost effectiveness evaluations for MAS is $26.88 rather than $29.56 as a result of the decision tree modelled analysis that considers the proportion of patients in each arm receiving an outcome instead of the mean costs stated above. Similarly, UC is $19.75 instead of $22.28

### Sensitivity analyses

#### Deterministic SA

**Fig. 2:**
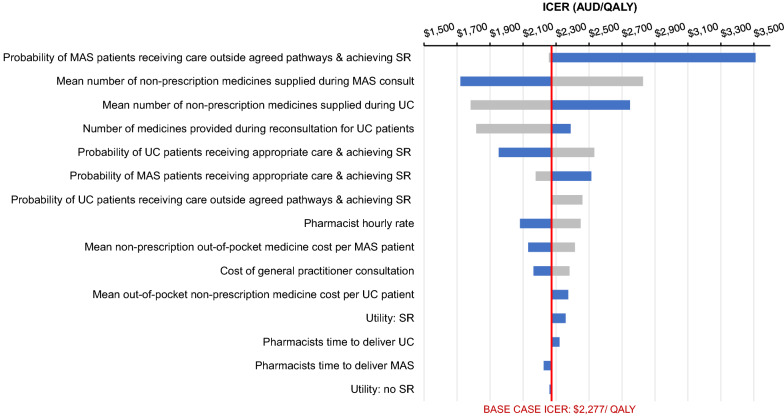
ICER tornado diagram for multiple one-way SA. *AUD* Australian dollars, *ICER* incremental cost effectiveness ratio, *MAS* minor ailment service, *QALY* quality adjusted life year, *SR* symptom resolution, *UC* usual pharmacist care. Grey indicates a lower value for each variable was applied. Blue indicates a higher value for each variable was applied. Red indicates the base case ICER value

The tornado graph displays bars for each parameter depicting which variables (greatest to smallest) impacts the estimated mean ICER (Fig. 2). The variable with the greatest impact on the ICER result was the probability of MAS patients receiving pharmacist care outside the agreed pathways and achieving symptom resolution. The mean number of medicines supplied during a MAS consultation was the variable with the second greatest impact on results. The impact on the ICER was almost null when the parameters of training costs and the average duration of consultation were changed.

The results of the multi-way SA are found in Table 6.

**Table 6:** Multi-way sensitivity analysis

	Highest mean cost per patient	Total outcome	Inc. cost (AUD)	Inc. outcome	ICER (AUD/outcome)
Outcome = QALY					
UC	$22.86	0.0264			
MAS	$33.84	0.0296	$10.98	0.003	$3502

*AUD* Australian dollars, *ICER* incremental cost effectiveness ratio, *MAS* minor ailment service, *QALY* quality adjusted life year, *UC* usual pharmacist care

#### Probabilistic SA

**Fig. 3:**
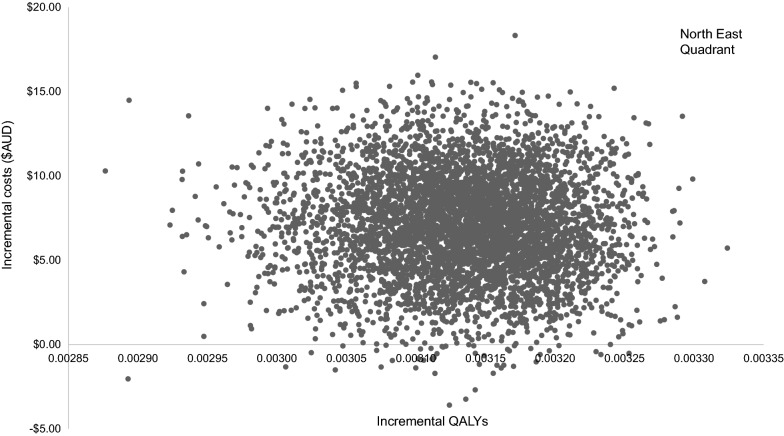
Cost effectiveness plane for MAS over UC. *AUD* Australian dollars, *ICER* incremental cost effectiveness ratio, *MAS* minor ailment service, *QALY* quality adjusted life year, *UC* usual pharmacist care

The results of 5000 simulations were found to produce stable results and are presented in a cost-effectiveness plane (scatter plot) (Fig. 3). Each iteration (point on the graph) represents an incremental cost and incremental benefit when model parameters take random values from a pre-specified range and probability distributions (full details of the analysis are provided in Additional file 1). The area to the right of the vertical axis is clinically beneficial, while the area above the horizontal axis is cost-increasing. Therefore, iterations are primarily in the north-east quadrant of the plane, reiterating MAS is more costly and more effective, than UC.

**Fig. 4:**
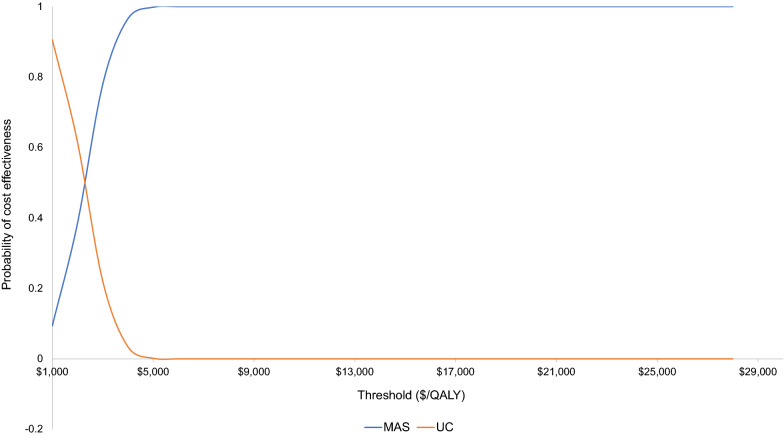
Cost effectiveness acceptability curve showing the probability of MAS being cost-effective at different willingness-to-pay thresholds. *AUD* Australian dollars, *ICER* incremental cost effectiveness ratio, *QALY* quality adjusted life year

The probability of MAS being cost-effective for a range of willingness-to-pay thresholds is presented in a cost-effectiveness acceptability curve (CEAC) (Fig. 4). The CEAC shows that MAS has a probability of being cost-effective from 9% at a willingness-to-pay of $1000 per QALY to 100% at a willingness to pay of $6000 per QALY, compared with UC. The probability that the intervention was cost-effective at the recommended threshold of $28,033 per QALY was 100%.

## Discussion

MAS has already proven clinical effectiveness when compared to current usual practice in Australian community pharmacies [34, 35]. This study provides details of a CUA evaluating MAS compared to UC, which was undertaken from a societal perspective. The robustness of results and uncertainty in model parameters was addressed by conducting a series of sensitivity analyses. The results indicate an ICER of $2277 (95% CI 681.49–3811.22) per QALY indicating higher costs and QALYs with MAS compared to UC. Based on a reference threshold of $28,033/QALY, findings suggest that implementation of MAS in Australian community pharmacies is a value for money intervention. The results presented should be interpreted and compared to ICERs from previous studies of health services that were accepted (or not) at clinical and policy levels within the Australian setting [47].

### Comparison to international literature

A number of countries have adopted MAS models. However, the international literature primarily provides comparison of pharmacy based minor ailment care to other health settings, such as general practice and ED. These studies suggest that the implementation of MASs leads to more efficient use of GP and ED services and overall health spending [48–53]. In the UK, Watson et al., estimated the cost and effectiveness (patient health outcomes) of pharmacy-based care for minor ailments compared with care provided by general practitioners or in ED settings [25]. Mean overall costs per consultation were £29.30 for pharmacy-based care compared with general practice (£82.34) and ED (£147.09) [25]. Pharmacy-based care for minor ailments was estimated to be less costly and as effective (in terms of symptom resolution) compared with ED and general practice [25]. Authors concluded that pharmacy-based minor ailment care was the dominate option [25]. Similarly in Canada, Rafferty et al. conducted an economic impact analysis measuring costs of a minor ailment program and the alternative scenario of UC, using primary data on pharmacists’ prescribing consultations in Saskatchewan [16]. After 5 years of implementation, cumulative cost savings were projected to be $3482,660 Canadian dollars (CAD), from a societal perspective. The study identified the cost to deliver the service at $18/consult (CAD), when compared with the cost of a GP consultation ($66.40 CAD) or an ED visit ($138 CAD) [16]. Comparatively, our study findings revealed a mean cost per MAS consult to be $29.56 AUD, compared to $22.28 for a pharmacist to conduct UC. Though, the variability in conditions considered as minor ailments in the literature represents a challenge for comparison of our results and data interpretation [54]. Studies identified in the international literature do not use a randomised trial study design, utilise comparators other than usual care, and in some instances do not specify the member of staff involved in providing management. Future research for MASs implemented within international health systems would be valuable. This should include clinical and economic data with a comparator of usual care.

### Strengths and limitations

There are some limitations to our study. While the decision tree model is a step forward in mapping minor ailment interactions and their implications, it is a simplification of reality and is subject to the trade-offs between data availability and assumptions made in constructing the model. We treated our study population as a full cohort and have assumed patients lost to follow-up behave similarly (i.e. similar probability of adhering to referral advice or reconsulting within 14 days) and their health status resolves (i.e. similar probability of achieving symptom resolution) in a similar way to patients followed-up. Our cRCT study was powered to detect changes in the outcome of ‘appropriate pharmacist care’ and this was assumed to lead to changes in symptom resolution. By definition, a minor ailment is self-limiting and involves symptom resolution regardless of pharmacist (or medical) intervention. Given symptom resolution probabilities were incorporated into the model, this impacts the results of our economic evaluation. While we saw a positive effect on resolution rates with MAS, the differences in symptom resolution were small compared with UC (RR 1.06; 95% CI 1 to 1.13; p = 0.035 [29]). This is reflected in an additional sensitivity analysis undertaken whereby MAS resulted in an ICER of $586.88 per extra patient achieving symptom resolution.

A time horizon of 14-days was considered appropriate to account for costs and health outcomes for the conditions evaluated (e.g. the common cold would normally resolve in 7–10 days). A 14-day time horizon has been previously applied in international studies assessing minor ailments and symptom resolution rates [25]. The timeframe was also chosen by researchers to reduce the possibility of recall bias [30]. It is important to acknowledge that conditions such as migraine, low back pain, reflux and dysmenorrhoea may be episodic or self-limiting, however also may be recurrent or chronic in nature. Methodological issues regarding time will have implications when evaluating the cost-effectiveness of healthcare services [55]. Analyses with shorter time horizons potentially omit relevant outcomes and therefore may provide incorrect results [55].

Utility values were not available from our cRCT study data, hence we relied on utility values reported in published literature [25]. The transferability of utility scores between jurisdictions remains unclear and the utility weights applied may not represent Australian preferences. A literature review by Knies et al. [56] discussed the international transferability of utilities derived from EQ-5D questionnaires. The authors found differences between national EQ-5D value sets and discouraged the application of utilities from other countries [56]. Although this is acknowledged as an important limitation, the use of literature estimates was considered the best available evidence to conduct the CUA. Furthermore, the use of QALYs to capture health benefits in a short time horizon (14 days) is also contentious and poses challenges when interpreting results. However, the use of QALYs were considered appropriate mainly because they provide a common metric for comparing cost per unit of health gain and this is currently the outcome measure recommended in Australia. It was not possible to capture the likely gradual increment in QALY as we didn’t have trial or published data to rely on. The impact of assuming a direct QALY gain is that the total accrued QALYs may be slightly overestimated. Though, because this assumption is occurring in both arms, it is unlikely to impact overall results. We attempted to improve the transferability of results to wider Australia using nationally reported unit costs and accounted for potential variation in costs through SA.

Local variation in practice, for example referral rates to general practice, can greatly influence the cost of providing MAS. This was evidenced in our clinical evaluation findings [29] which identified that MAS pharmacists referred four times as many patients when using the agreed clinical pathways, compared with UC. The high referral rate was a result of patients identified to be self-medicating or experiencing symptoms for prolonged periods without assessment or re-assessment by a medical practitioner [29]. Furthermore, patients receiving MAS were five times more likely to follow through with referrals made by the pharmacist during the consultation, compared to current practice [29].

The extent of transferability of findings is dependent on the context of design and implementation. A methodological consideration is the urban Australian community pharmacy setting this study was conducted. Future studies to confirm or enhance implementation of MAS in other contexts would help address these limitations. Furthermore, refining the decision tree model by addressing some of its limitations or confirming transition probabilities in future evaluations would be useful to validate the economic findings in this study.

### Implications for policy and practice

This research was conceived and undertaken at a time of change to the health landscape in Australia [57]. The Australian health system is faced with challenges of improving accessibility and quality of care in the face of constrained funding [57]. Policy makers, at governmental and organisational levels, are increasingly interested in cost-effective, evidence-based, patient-centered services. The drivers of this interest are equally to save the health system money, improve patient outcomes and quality use of medicines.

Australian primary care will need to undergo reforms that incentivise pharmacists to deliver self-care effectively. International models of community pharmacy care exist that attempt to address these challenges. Scotland, for example, uses a capitation model of remuneration and provides pharmacists with the responsibility for the care of individual patients registered to that pharmacy [58].

It is recommended that due consideration be given for community pharmacies nationwide to adopt and implement MAS. There should be a focus on upskilling community pharmacists to deliver MAS in an integrated and coordinated capacity. Policy and funding alignment will also be a major determinant for future sustainability. Expanding community pharmacists’ scope through training, as seen in the UK and Canada, for other clinical areas such as minor abrasions, wounds, strains and sprains, minor burns etc. or prescribing of certain prescription medicines within a collaborative model for certain conditions is likely to add further economic benefits.

## Conclusion

There is significant potential to amplify self-care and responsible self-medication in Australia. With national implementation in the Australian health system there is potential for system efficiency gains, demonstrated through delivering health care that is optimally cost-efficient and clinically effective at an appropriate level, and working collaboratively within an integrated system. The implicit assumption is that patients consulting GPs or EDs for these conditions could be reduced by transferring patients, where appropriate, to the community pharmacy setting with the aim of fully utilising primary health locations and professionals in Australia.

## Supplementary information

**Additional file 1.** Details of the probabilistic sensitivity analysis.

## Data Availability

The datasets used and analysed during the study are available from the corresponding author on reasonable request.
