# Supplementary material for: Cost utility of a pharmacist-led minor ailment service compared with usual pharmacist care
Source: Cost Eff Resour Alloc. 2020 Jul 28;18:24. doi: 10.1186/s12962-020-00220-0 (PMC7388462; doi:10.1186/s12962-020-00220-0)
Supplement: Supplementary file 1 — Additional file 1. Details of the probabilistic sensitivity analysis. [file 12962_2020_220_MOESM1_ESM.docx]

**Additional File 1 Details of the probabilistic sensitivity analysis**

| Health resource | Mean model value | Std. error | Minimum | Maximum* | Alpha | Beta | Parametric distribution |
| --- | --- | --- | --- | --- | --- | --- | --- |
| Costs | | | | | | | |
| Pharmacist rate (per hour) | $29.37 | $2.52 | $24.04 | $34.30 | $136.34 | $0.22 | Gamma |
| Time to deliver MAS (minutes per patient) | 10.88 | 0.18 | 10.52 | 11.23 | 10.88 | 2.73 | Normal |
| Time to deliver UC (minutes per patient) | 3.29 | 0.21 | 2.88 | 3.71 | 3.29 | 0.79 | Normal |
| Trainings with MAS (number per year) | 1 | 0.51 | 0 | 2 | 1 | 0.12 | Normal |
| Facilitator rate with MAS (per hour) | $46.28 | $4.72 | $37.02 | $55.54 | $96.04 | $0.48 | Gamma |
| Facilitator visits with MAS (per month) | 1 | 0.51 | 0 | 2 | 1 | 0.12 | Normal |
| Average training, facilitation and technology cost with MAS (per patient) | $0.07 | $0.02 | $0.00 | $0.11 | $15.37 | $0.00 | Gamma |
| Average nonprescription medicine price with MAS (per patient) | $10.62 | $0.22 | $10.20 | $11.05 | $2,364.01 | $0.00 | Gamma |
| Average nonprescription medicine price with UC (per patient) | $9.76 | $0.20 | $9.39 | $10.14 | $2,494.37 | $0.00 | Gamma |
| Average cost of medicines at reconsult (per patient) | $9.79 | $0.94 | $7.94 | $11.64 | $108.11 | $0.09 | Gamma |
| General practitioner fee (per consult) | $44.07 | $6.74 | $30.85 | $57.29 | $42.68 | $1.03 | Gamma |
| Utilities | | | | | | | |
| Symptom resolution | 0.91 | 0.02 | 0.88 | 0.94 | 327.03 | 32.34 | Beta |
| No symptom resolution | 0.77 | 0.02 | 0.73 | 0.81 | 421.87 | 126.01 | Beta |

***Abbreviations:*** *MAS: Minor ailment service; UC: usual pharmacist care.*

** Lower and upper bound values represent 95% confidence interval; or upper and lower range from trial data.*
